# Use of Barbed Sutures in Endoscopic-Assisted Septal Surgery: A Technical Report of a Knot-Free Technique

**DOI:** 10.7759/cureus.78006

**Published:** 2025-01-26

**Authors:** Simon Morris, Paramesh Puttasiddaiah, Heikki Whittet

**Affiliations:** 1 Otolaryngology, Morriston Hospital, Swansea, GBR

**Keywords:** barbed sutures, deviated nasal septum, endscopic septoplasty, nasal endoscopic surgery, suture techniques

## Abstract

Nasal septal surgery is routinely undertaken in patients who have septal deviation causing nasal obstruction. This manuscript describes our use of absorbable, knotless, looped, unidirectional barbed suture materials in patients undergoing endoscope-assisted septal surgery. Barbed sutures do not require sutures to be tied within the nasal cavity, which saves time intra-operatively and prevents the loosening of sutures post-operatively. Our experiences demonstrate the benefits of this novel technique over traditional septal quilting suture methods, and it has become standard practice within our unit for endoscope-assisted septoplasty.

## Introduction

Nasal septal surgery is routinely undertaken in patients who have septal deviation causing nasal obstruction or for access in patients undergoing other endonasal procedures [1]. Over the years, this common surgery has evolved to become minimally invasive with various techniques described in the literature, including endoscope-assisted septal surgery. Endoscope-assisted septoplasty has some advantages over traditional headlight septal surgery, where the dissection can be limited to the area of deviation, reducing the size of flaps and morbidity of post-operative swelling [2]. Nonetheless, mucosal flaps still require quilting sutures to prevent complications such as synechiae, septal hematoma, and epistaxis. This can be technically challenging when performed endoscopically. Several surgical techniques have been offered as a solution for this in the literature, including septal splints or staplers [3]; however, this technical report demonstrates the use of barbed sutures.

Barbed sutures have widespread use in general, gynecological, and plastic surgery, including descriptions in septorhinoplasty [4,5]. Within otolaryngology, they have been used in pinnaplasty to good effect [6]. These are monofilament sutures with multiple tiny “barbs”, which can be both unidirectional and bidirectional to the needle tip, and prevent the suture from sliding backward. They are also “looped”, which negates the need for a suture knot to be thrown at depth, potentially saving intra-operative time and negating the complications with loosening of quilting suture.

This manuscript describes the technical feasibility and methodology of using barbed sutures in patients undergoing endoscope-assisted septal surgery at a single institution.

## Technical report

In this single Ear, Nose, and Throat (ENT) unit, barbed sutures have been used for patients undergoing endoscope-assisted septal surgery to quilt the nasal septal mucosa since 2021. The knotless septoplasty technique can be utilized for any patient undergoing endoscopic septoplasty, either as primary nasal septal corrective surgery or as part of a wider procedure for access.

There are two absorbable, knotless, looped, unidirectional barbed suture materials currently on the market: the Stratafix^TM^ Symmetric Polydioxanone (PDS) Plus Knotless Tissue Control Device (Ethicon, USA) and V-Loc^TM^ Wound Closure Device (Medtronic Covidien, USA).

As demonstrated in Video 1, the procedure involves passing the needle through and through the posterior septal flap to the contralateral nasal cavity and then reversing it through to the anterior septal flap. Further, the needle is passed through the loop and tightened to appose the mucosal flaps. This process is repeated, and the suture is cut. This leaves an ‘X’ appearance of the suture material across the mucosal incision and a free edge to the suture on the contralateral side. It does not slip due to the unidirectional suture barbs. Patients are then discharged with the instruction to use an emollient ointment in the nasal cavity twice daily for two weeks post-operatively. As with any septal surgery, it is imperative that mucosal flaps are handled with care intra-operatively and maintained to prevent the risk of septal perforation.

**Video 1:** Demonstration of the technique for barbed suture septoplasty

Results

A total of 50 patients underwent endoscope-assisted septoplasty with barbed quilting suture in the three-year period. No cases of septal hematoma, perforation, or suture granuloma were noted at the three-week post-operative review (n=0). All patients were reviewed three months post-operatively, where all outcomes were recorded before being discharged as part of our standard post-operative regimen.

On some occasions, the free edge of the suture material was left too long, which may cause irritation to the lateral nasal wall and give patients a sensation of foreign body in the nose. This can be difficult to predict intra-operatively when there is an element of mucosal edema; as such, this free suture edge may require trimming in the first post-operative review if the suture material has not already dissolved. No cases of suture material loosening were observed due to the knot-free and unidirectional nature of the suture material. 

## Discussion

Endoscope-assisted septal surgery offers clear visualization of the septum, particularly posterior spurs, and permits careful dissection of the submucosa to prevent iatrogenic injury, as well as enhanced training opportunities [1-3]. In our experience, this further aids placement of the looped barbed suture, ensuring adequate placement intra-operatively, avoiding iatrogenic trauma when tying knots within the nasal cavity, and avoiding over- or under-tightening of sutures, which may cause morbidity such as pressure necrosis or suture extrusion. In addition, the certainty of suture placement can negate the need for nasal packs, which is associated with improved patient outcomes [7].

This technique also has potential advantages relating to intra-operative time and training purposes for residents, where tying knots at depth during septoplasty can be a challenging skill to both learn and teach during conventional headlight-septal surgery [8]. It also provides a valuable alternative option to the established techniques using septal splints, packs, or septal staplers [3].

This manuscript purely reports the technical ability and methodology for the performance of this technique. It is limited by the lack of objective outcomes or direct comparison to existing quilting techniques. Further research is planned to provide objective and subjective outcome measures (including intra-operative ease, time, and post-operative outcomes) in order to determine the value of the wider use of these suture materials in endoscope-assisted septal surgery. This technical report will form the basis for further study within this unit to determine this technique's value as an alternative septal quilting method. 

## Conclusions

This is the first known description of barbed sutures in endoscope-assisted nasal septal surgery. Our study demonstrates the benefits of this novel technique over traditional septal suture methods. Quilting the septal mucosa during endoscope-assisted septoplasty can be a challenging activity due to the “one-handed” technique required; however, the barbed suture allows a no-tie technique, with no post-operative suture loosening, and has potential advantages for trainees learning to perform septoplasty quilting suture.
